# Concurrent short-term use of prednisolone with cyclosporine A accelerates pruritus reduction and improvement in clinical scoring in dogs with atopic dermatitis

**DOI:** 10.1186/1746-6148-9-173

**Published:** 2013-09-03

**Authors:** Ramiro Dip, James Carmichael, Ingrid Letellier, Guenther Strehlau, Elizabeth Roberts, Emmanuel Bensignor, Wayne Rosenkrantz

**Affiliations:** 1 Novartis Animal Health Inc., Schwarzwaldallee 215, CH-4058 Basel, Switzerland; 2 Institute of Pharmacology and Toxicology, University of Zurich-Vetsuisse, Winterthurerstrasse 260, CH-8057 Zurich, Switzerland; 3 Novartis Animal Health US, Inc., 3200 Northline Ave., Ste 300, NC 27408 Greensboro, USA; 4 Novartis Animal Health S.A.S., 14 Boulevard Richelieu, Boite Postale 430F-92845 Rueil-Malmaison Cedex, France; 5 Veterinary Dermatology Referral Service, 6 rue de la Mare Pavée, F-35510 Cesson Sevigne, France; 6 Animal Dermatology Clinic, 2965 Edinger Ave., CA 92780 Tustin, USA

**Keywords:** Cyclosporine, Ciclosporine, Atopic dermatitis, Pruritus, Prednisolone, Clinical safety, Atopica

## Abstract

**Background:**

A randomized, unmasked, multicenter study was conducted to evaluate the rate of pruritus reduction and improvement in clinical scoring by cyclosporine A (5 mg/kg orally, once daily for 28 days) either alone (n = 25 dogs) or with concurrent prednisolone (1 mg/kg once daily for 7 days, followed by alternate dosing for 14 days; n = 23 dogs) for the treatment of atopic dermatitis in dogs. Dogs were included in the study after exclusion of other causes of pruritic dermatitis, and were assessed by dermatologists on days 0, 14 ± 1 and 28 ± 2. Assessments included: general physical examination, CADESI-03 lesion scoring, overall clinical response, evaluation of adverse events (AEs), body weight and clinical pathology (hematology, clinical chemistry and urinalysis). Owner assessments, including pruritus (visual analogue scale, VAS) and overall assessment of response were conducted every 3–4 days, either during visits to the clinic or at home. Owners reported AEs to the investigator throughout the study.

**Results:**

By day 28 ± 2 both treatment groups resulted in a significant improvement of the atopic dermatitis. Both investigators and owners agreed that concurrent therapy resulted in a quicker improvement of the dogs ‘overall’ skin condition and of pruritus (significant reduction of pruritus by day 3–4, 72.8% improvement by day 14 ± 1), when compared to cyclosporine A alone (significant reduction of pruritus by day 7–8, 24.7% improvement by day 14 ± 1). CADESI-03 scores significantly improved in both groups by day 14 ± 1 onwards, and there were no significant differences in the scores between treatment groups at any time points. A total of 56 AEs (cyclosporine A alone = 34; concurrent therapy = 22) were reported in 33 dogs. No dogs died or stopped treatment due to an AE. The most commonly reported AEs in the cyclosporine A group were associated with the digestive tract, whilst systemic disorders were reported more frequently observed following concurrent therapy. Evaluation of body weight change and clinical pathology indices showed no overall clinically significant abnormalities.

**Conclusions:**

In dogs with atopic dermatitis, a short initiating course of prednisolone expedited the efficacy of cyclosporine A in resolving pruritus and associated clinical signs. The observed adverse events were consistent with those expected for the individual veterinary medicinal products.

## Background

Atopic dermatitis, a common skin disease of dogs, is defined as a genetically predisposed inflammatory and pruritic skin disease with characteristic clinical features associated with IgE antibodies, usually directed against environmental allergens [1,2]. The chronic form of the disease is characterised by pruritus with or without recurrent skin or ear infections. Primary skin lesions usually consist of erythematous macules, patches and small papules. Most patients, however, present with lesions that occur secondary to self-trauma, including self-induced alopecia, lichenification and hyperpigmentation. In addition to measures such as allergen avoidance and improved skin and coat hygiene, pharmacological agents are often necessary to manage the intensity of pruritus, which has an impact on the perceived quality of life of both owner and dog [3].

The 2010 practice guidelines issued by the International Task Force on Canine Atopic Dermatitis (now the International Committee on Allergic Diseases of Animals) concluded that there is good evidence for the high efficacy of both oral cyclosporine A and oral glucocorticoids such as prednisolone, as a treatment for chronic atopic dermatitis in dogs [4]. Both are equally effective treatments but have different modes of action and clinical response profiles [5]. Cyclosporine A, a cyclic oligopeptide macrolide, blocks the activity of cytoplasmic calcineurin phosphatase thereby specifically inhibiting cytokine induced activation of cells that initiate the cutaneous immune response and mediate allergic reactions. Glucocorticoids bind to the cytosolic glucocorticoid receptor, thereby regulating cellular gene expression and protein transcription, exerting a wide variety of systemic effects including the anti-inflammatory action which helps to reduce pruritus. Glucocorticoids, such as prednisolone when dosed at 0.5 mg/kg twice daily, have a rapid onset of clinical activity; cyclosporine A requires approximately four weeks of therapy (5 mg/kg once daily) for maximal clinical improvement, before the dose may then be reduced [6]. Undesirable effects of chronic oral glucocorticoid therapy (e.g. polyuria, polydipsia, polyphagia, predisposition to urinary tract and skin infections) are common, but even short term use can have systemic consequences such as urinary incontinence and lethargy [5,7]. Common adverse events after initiating cyclosporine A therapy (e.g. vomiting, diarrhoea) generally improve spontaneously upon further administration [6]. For these reasons, together with its more targeted action, cyclosporine A is an excellent treatment option for life-long therapy of chronic atopic dermatitis in dogs [6,8].

Prolonged administration of elevated doses of glucocorticoids to dogs can result in immunosuppressive effects (recently reviewed by Whitley NT and Day J [9]). Similarly, administration of cyclosporine A at doses higher than those recommended for the treatment of atopic dermatitis have been used for the treatment of immune-mediated disease [10,11]. Therefore, the concurrent long-term administration of oral cyclosporine A and glucocorticoids is not recommended, as potential combined immune suppression could result in a higher risk for development of potentially severe opportunistic infections of the skin or other organs. However, the administration of a short course of oral glucocorticoids during the first 2 weeks of cyclosporine A administration has been suggested as a therapeutic strategy to accelerate the improvement of pruritic signs of atopic dermatitis [4]. To the best of our knowledge, this treatment regimen has not previously been assessed under controlled conditions. Therefore, in this study both the efficacy and safety of such a regimen were evaluated in a multicenter clinical trial.

## Methods

### Study design

This was a randomized multicenter field study designed to compare the safety and the rate of improvement of pruritus reduction and other signs of atopic dermatitis in dogs by cyclosporine A alone or with the concomitant administration of prednisolone during the initiation of cyclosporine A therapy. Neither investigators nor owners were blinded to treatment. The study was conducted with board certified dermatologists in five clinics in France and five in the United States, in consideration of the following: VICH GL9 on Good Clinical Practice, 15^th^ June 2000; Directive 2001/82/EC of the European parliament, 6^th^ November 2001: The Community Code relating to veterinary medicinal products; EMEA/CVMP/816/00, guideline to the statistical principles for veterinary clinical trials – 5^th^ June 2002. Permits to conduct this study were not required in either country, since this was a study conducted with authorized products within current product labeling. Owner consent was obtained prior to screening any dog for the study.

### Animals

Client-owned dogs (≥6 months of age) showing clinical signs of atopic dermatitis and an owner assessed pruritus visual analogue scale (VAS) score of at least 50% were included in the study. All dogs had received adequate flea treatment as determined by the investigators for at least four weeks prior to inclusion in the study, had completed an elimination diet trial, had no active bacterial or fungal infection, and fulfilled at least five of Favrot’s 2010 criteria [12], confirming the diagnosis of atopic dermatitis. Dogs were otherwise considered to be in good health, as determined by a comprehensive physical examination and clinical pathology (hematology, clinical chemistry, urinalysis). Any concomitant treatment which could interfere with the assessment of efficacy or safety at any time prior to or during the study was not permitted (see Table 1). Dogs were kept in their home environment, received an adequate flea treatment throughout the study and were managed in their usual way, without any changes in diet during the study.

**Table 1:** Prohibited concomitant medications

**Treatments prohibited within 7 days of starting the study**	
● Any antibiotic	● Topical corticosteroids
● Drugs interfering with CsA such as ketoconazole, itraconazole, miconazole, phenobarbital.	
**Treatments prohibited within 14 days of starting the study**	
● Cylosporine A	● Systemic short acting glucocorticosteroids, including ophthalmic and otic preparations
● Antihistamines	● Non-steroidal anti-inflammatory drugs (NSAIDs)
● Topical calcineurin inhibitors	● Shampoos (except where the same treatment regimen is maintained throughout the study).
● Vaccination	● Clomipramine, amitriptyline and fluoxetine and any other serotonin reuptake inhibitor
**Other prohibited treatments**	
● Allergen specific immunotherapy (except if initiated for at least 9 months and where the same treatment regimen is maintained throughout the study).	
● Treatment with systemic long-acting corticosteroids within the last 3 months	
● Essential fatty acids except those used before study initiation for at least 57 days and where the same treatment regime is maintained throughout the study	

### Treatment administration

**Figure 1:**
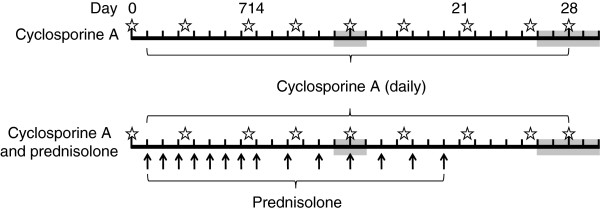
**Study design.** Dogs were included in the study on day 0 and received one daily treatment with cyclosporine A (Atopica®, 5 mg/kg) from day 1 to day 28. In addition dogs in the cyclosporine A and prednisolone group received 1 mg/kg prednisolone once daily, on the indicated days (arrows). Grey boxes represent clinic visit windows (day 14 ± 1 and day 28 ± 2) and stars represent owner assessment time points.

At inclusion (day 0), dogs were randomized to one of the treatment groups following site specific randomization lists prepared with SAS®, version 9.2 (2008) (SAS® 9.2 Help and Documentation, Cary, NC, SAS Institute Inc., Copyright 2010), which were not separated for sexes, age or body weight, and was equally balanced for both treatment groups. Dogs initiated treatment on day 1 with cyclosporine A (Atopica®, Novartis Animal Health Inc., Basel, Switzerland) at approximately 5 mg/kg orally once daily for 28 (±2) days either alone (treatment group 1; n = 25) or concomitantly (treatment group 2; n = 23) with prednisolone (Megasolone®, Merial (France); Prednisolone®, Vedco (USA)) at approximately 1 mg/kg orally once daily for seven doses (day 1 through 7), followed by approximately 1 mg/kg orally every other day for a further seven doses (day 8 through 20). The treatment schedule is summarized in Figure 1. Owners kept a record of treatment administration; all drugs dispensed to and returned from the owner were accounted for at the end of the study.

### Observations

Dogs were evaluated by both the owner and the investigator in the clinic on the day of enrollment (clinic visit 1, day 0), after 2 weeks (clinic visit 2, day 14 ± 1) and after 4 weeks (clinic visit 3, day 28 ± 2) of treatment. Owner evaluation included an assessment of pruritus with a visual analogue scale (VAS, [13]) and overall response at day 14 ± 1 and day 28 ± 2 (Table 2). In addition, on three separate occasions during each 2 week period between the scheduled clinic visits, the owner assessed pruritus and the overall response of the dog in its own environment. Throughout the study, the owner documented and reported all adverse events (AEs) to the investigator.

**Table 2:** Efficacy end-points

**Assessment**	**Type of assessment**	**Criteria**
**Owner assessments**		
Pruritus (Primary endpoint)	Pruritus VAS	0 = “my dog is not itchy: no scratching, chewing, rubbing or licking observed”
		100 = “my dog is extremely itchy: scratching, chewing, rubbing or licking constantly”
		Individual pruritus observations on VAS at any time were expressed as a percentage:
		*VAS_P = 100% × VAS length / total length of scale*[13]
Overall assessment of clinical response	Score	[0] *Poor:* My dog’s dermatological condition has deteriorated or not changed compared to before treatment.
		[1] *Moderate:* My dog’s dermatological condition has only slightly improved compared to before treatment.
		[2] *Good:* My dog’s dermatological condition has clearly improved compared to before treatment.
		[3] *Excellent:* My dog’s dermatological condition has completely recovered as compared to before treatment.
**Clinical assessments**		
Overall assessment of clinical response	Score	[0] *Poor:* worsening or no change of the clinical signs of the atopic dermatitis as compared to initial examination.
		[1] *Moderate*: slight amelioration of the clinical signs of the atopic dermatitis as compared to initial examination.
		[2] *Good:* clear amelioration of the clinical signs of the atopic dermatitis as compared to initial examination.
		[3] *Excellent:* Clinical signs of the atopic dermatitis observed during the first examination have completely disappeareds.
CADESI-03	Score	Completion of validated template grading erythema, lichenification, excoriations, alopecia and the sum score for all four lesions at 62 body sites (grades, none: 0; 1: mild; 2, 3: moderate; 4, 5: severe) [13].

Clinical evaluation by the investigator at each clinic visit included: general physical examination, including body weight, CADESI-03 lesion scoring according to Olivry et al. [13], overall clinical response and assessment of AEs (Table 2).

Any observation in a treated dog that was unfavorable and unintended and occurred after treatment administration, was considered an adverse event whether or not considered to be product related, according to the VICH Guideline 9 [14]. All AEs were described in detail by the investigator and reported to the relevant regulatory authorities in accordance with local regulations.

Blood and urine samples were collected within 14 days prior to inclusion (pre-inclusion screening visit) and on visit 3 (study completion, day 28 ± 2). Complete hematological, clinical chemistry and urinalysis panels were determined.

### Statistical analysis

All statistical analyses were performed with the commercial software package SAS®, Version 9.2 (2008). The experimental unit was the individual dog, and unless otherwise indicated, the level of significance for all two-sided tests performed was α=0.05. The SAS® procedure *npar1way* was used to perform the Mann–Whitney U tests and procedure *univariate* was applied in order to calculate summary statistics, for test of normality (Shapiro-Wilk test) and Wilcoxon paired sample tests. SAS® procedure *mixed* was applied to perform analyses of variance. SAS® procedure *freq* was applied to calculate contingency tables and to perform Fisher’s Exact tests. Summary statistics (arithmetic mean, minimum, maximum, median and standard deviation) were provided for all continuous parameters of interest.

#### Efficacy analysis

For the assessment of pruritus, non-parametric Mann–Whitney tests were applied for the comparisons of groups at various assessment time points and the percentage change in pruritus relative to untreated levels was calculated. Bonferroni corrections were applied for the adjustment of the effect of multiple testing. Overall clinical responses assessed by the investigators were compared with a Fisher’s exact test. For each group CADESI-03 lesion scores changes from baseline were compared with the Wilcoxon test, including Bonferroni corrections. In addition, a repeated measures model of covariance (RMANCOVA) was employed for evaluating the time by treatment interaction on CADESI-03 scores (normal distribution assumptions for CADESI-03 scores were partly satisfied after square root transformation). For normalization of the sum of CADESI-03 scores across groups, the arithmetic mean for each group at day 0 was considered as 100% and scores at each time-point were normalized by multiplying the individual score by 100 and dividing by the treatment group arithmetic mean at day 0.

The level of significance for all parameters was 0.05, except for pruritus assessed by the owner (primary efficacy end-point). For this endpoint an unplanned interim analysis was performed and, therefore, the significance level was adjusted to 0.025 to maintain experiment wise type 1 error rate.

#### Safety analysis

Adverse events and associated clinical signs were summarised and compared between treatment groups. Mann–Whitney tests were applied for the various comparisons between groups. Wilcoxon paired sample tests were additionally applied to test for changes of clinical pathology parameters from baseline separately within each group. Comparisons of groups with respect to ‘at least one adverse event’ versus ‘no adverse event’ were performed with Fisher’s Exact tests.

## Results

### Study population

Forty-eight dogs (24 males and 24 females) of 1.25 – 12.4 years of age were enrolled in France (n = 23) and in the United States (n = 25). The study population consisted of 41 pure breed dogs representing 22 breeds, plus 7 mixed breed dogs. The population was evenly distributed between groups, with no significant differences for any demographic variables except for mean body weight: dogs randomized to receive cyclosporine A alone had a significantly lower pre-treatment body weight than those randomized to concurrent therapy (16.93 kg vs. 28.66 kg, respectively: p = 0.0012); this difference remained unchanged throughout the study.

Four cases received repeated incorrect doses and were therefore excluded from the efficacy analysis (one dog was under-dosed with cyclosporine A for the first 12 days of the study, 2 dogs were under-dosed with cyclosporine A throughout the study, and 1 dog received prednisolone daily instead of every other day during the second week of treatment). All 48 cases were included in the clinical safety analysis (cyclosporine A alone group n = 25; concurrent treatment group n = 23). There were no withdrawals due to lack of efficacy or adverse events.

### Pruritus

**Figure 2:**
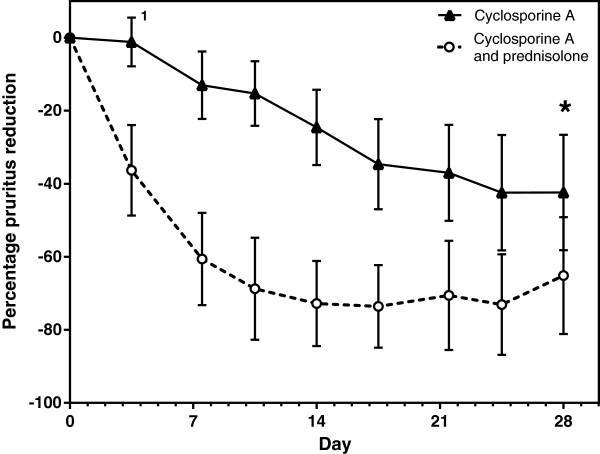
**Reduction of pruritus.** Mean percentage reduction in pruritus as assessed by the owner for dogs treated with cyclosporine A (▲) or with cyclosporine A and prednisolone (○). Bars indicate 95% confidence interval. At every time point after day 0 differences between both groups were statistically significant with the exception of day 28 ± 2 (_*_). Pruritus assessment by the owner at every time point was significantly lower than baseline for both treatment groups, with the exception of day 3 for the cyclosporine group (1, p = 0.3583).

For both treatment groups, owners reported a reduction in pruritus during treatment (Figure 2). By day 14 ± 1, dogs treated with cyclosporine alone showed 24.6% reduction, while dogs with concurrent treatment with prednisolone had an average pruritus reduction of 72.8% (Table 3). Differences in pruritus reduction between groups were significant for all time-points recorded with the exception of the final assessment (day 28 ± 2), in which pruritus reduction in dogs treated with cyclosporine alone was 42.4% compared with 65.1% in dogs with concurrent treatment (not significant, *p* >0.025).

**Table 3:** Owner-reported pruritus VAS scores and reduction from baseline following treatment

**Day**	**Treatment (n = 22)**	**Score**					**Score change from baseline (%)**	**p-value**
		**Mean**^**2**^	**Median**	**s.d.**	**Min**	**Max**	**Mean (95% CI)**	
0	Cyclosporine A	79.28	80.58	12.45	52.60	98.92	**-**	**-**
	Cyclosporine A and prednisolone	73.90	73.12	11.83	50.75	100.00	**-**	
3-4	Cyclosporine A	77.43	79.03	11.42	48.50	95.02	−1.18 (−7.8,5.5)	0.0008
	Cyclosporine A and prednisolone	47.65	50.00	22.86	10.95	96.79	−36.33 (−48.7,-24.0)	
7-8	Cyclosporine A	67.79	69.65	14.62	38.71	95.02	−13.04 (−22.3,-3.8)	0.0008
	Cyclosporine A and prednisolone	29.21	29.50	20.46	1.99	72.04	−60.61 (−73.3,-47.9)	
10-11	Cyclosporine A	65.81	67.99	13.47	36.56	87.06	−15.31 (−24.2,-6.5)	0.0008
	Cyclosporine A and prednisolone^**1**^	23.08	16.13	22.97	0.00	74.63	−68.73 (−82.7,-54.7)	
14 ± 1	Cyclosporine A	58.69	57.53	18.03	26.87	93.03	−24.57 (−34.9,-14.2)	0.0008
	Cyclosporine A and prednisolone	20.37	12.90	20.22	0.00	75.62	−72.79 (−84.4,-61.1)	
17-18	Cyclosporine A	50.45	47.54	19.71	20.40	88.56	−34.62 (−47.0,-22.3)	0.0008
	Cyclosporine A and prednisolone	19.33	10.75	19.50	0.00	70.00	−73.57 (−84.9,-62.3)	
21-22	Cyclosporine A	48.33	47.79	20.26	16.13	88.56	−37.00 (−50.1,-23.9)	0.0128
	Cyclosporine A and prednisolone	21.23	11.29	24.84	0.00	84.58	−70.55 (−85.5,-55.6)	
24-25	Cyclosporine A	43.69	42.36	24.29	3.23	88.56	−42.41 (−58.2,-26.6)	0.0240
	Cyclosporine A and prednisolone	19.90	10.75	24.42	0.00	93.03	−73.10 (−86.9,-59.3)	
28 ± 2	Cyclosporine A^**1**^	43.44	43.01	25.09	3.23	89.05	−42.36 (−58.2,-26.5)	0.2160
	Cyclosporine A and prednisolone	26.26	15.05	28.54	0.00	94.03	−65.12 (−81.1,-9.1)	

^**1**^n = 21; ^**2**^arithmetic mean; *s*.*d*. standard deviation.

When comparing to baseline, non-parametric pair-wise analysis of pruritus showed a significant reduction in VAS as assessed by the owner as early as day 3–4 in dogs treated with cyclosporine A and prednisolone (p = 0.0001), while in dogs treated with cyclosporine A alone, a significant improvement of pruritus could be observed from day 7–8 onwards (p = 0.0022). VAS pruritus scores at all other time points remained significantly lower than baseline for both treatment groups.

### Overall response

**Figure 3:**
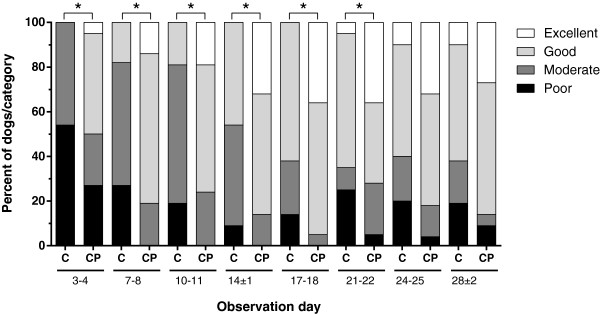
**Owner overall assessment of clinical response.** Percentage of poor, moderate, good and excellent responders among dogs treated with cyclosporine A (C) or cyclosporine A and prednisolone (CP) as assessed by the owner at the different observation dates. *: p ≤ 0.05 (Fisher's Exact, two-tailed).

Overall response assessments followed a similar trend as pruritus. Fifty percent of the dogs that received cyclosporine A with prednisolone had either a good or an excellent response on day 3–4 (Figure 3), while the percentage of dogs with either response increased to approximately 80% from day 7–8 onwards. In contrast, about 50% of the dogs treated with cyclosporine A alone had a moderate response on day 3–4. Response to this treatment improved thereafter, and by day 21–22 approximately 70% of the dogs in this treatment group had a good or excellent response.

For all assessments up to day 20–21 owners reported a significantly better overall response for concurrent therapy in comparison to cyclosporine A alone. For the last 2 assessments (day 24–25 and day 28 ± 2) the overall response was not significantly different between the groups. Significance values at all assessment points by the owners are summarized in Additional file 1: Table S1.

Investigators’ evaluations also indicated the overall clinical response to be significantly better for the concurrent therapy group at day 14 ± 1 (Table 4; p = 0.033). By day 28 ± 2, the observed treatment group differences were no longer significant (p = 0.4655).

**Table 4:** Overall clinical response: number (and percentage) of poor, moderate, good and excellent responders as assessed by the investigator

**Days**	**Response**	**Number of responders (%)**		**p-value**
		**Cyclosporine A (n = 22)**^**1**^	**Cyclosporine A and prednisolone (n = 22)**	
14 ± 1	Poor	4 (18.2)	1 (4.5)	0.0330
	Moderate	9 (40.9)	5 (22.7)	
	Good	8 (36.4)	8 (36.4)	
	Excellent	1 (4.5)	8 36.4)	
28 ± 2	Poor	4 (20)	2 (9.1)	0.4655
	Moderate	3 (15)	2 (9.1)	
	Good	11 (55)	12 (54.5)	
	Excellent	2 (10)	6 (27.3)	

^**1**^n = 20 for this treatment on day 28 ± 2.

### CADESI-03 scores

**Figure 4:**
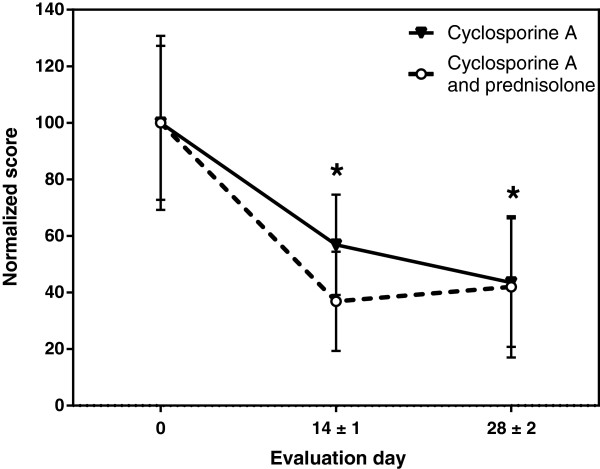
**Investigator assessment of skin lesions.** Sum of CADESI-03 scores, normalized. Mean and 95% confidence interval are presented for each evaluation day). *: p ≤ 0.05 at day 14 ± 1 and 28 ± 2 for both treatment groups against baseline (Wilcoxon paired sample test).

At both post treatment clinic visits, CADESI-03 scores for erythema, lichenification, excoriations, alopecia and the sum of all scores were significantly lower than pre-treatment values, for both treatment groups (Table 5 and Figure 4).

**Table 5:** CADESI-03 scores for the 4 lesions evaluated and sum of all scores by day of evaluation and treatment group

**Lesion**	**Day**	**Scores (s.d.) for the treatment groups**		p-value*
		**Cyclosporine A (n = 22)**^**1**^	**Cyclosporine A and prednisolone (n = 22)**	
Erythema	0	43.27 (29.8)	49.36 (26.81)	0.1185
	14 ± 1	27.14 (22.61)	20.36 (21.97)	
	28 ± 2	23.62 (33.17)	19.95 (19.47)	
Lichenification	0	17.23 (18.29)	23.23 (21.8)	0.6708
	14 ± 1	8.41 (9.03)	8.18 (11.17)	
	28 ± 2	6.86 (8.03)	9.55 (16.49)	
Excoriations	0	12.82 (15.53)	16.95 (17.61)	0.2552
	14 ± 1	4.36 (5.92)	4.05 (8.85)	
	28 ± 2	3.71 (5.44)	7.27 (16.67)	
Alopecia	0	20.00 (28.46)	20.32 (15.33)	0.1590
	14 ± 1	13.14 (14.27)	7.95 (10.17)	
	28 ± 2	6.38 (6.79)	9.45 (15.30)	
Sum of scores	0	93.32 (64.75)	109.86 (67.39)	0.0750
	14 ± 1	53.05 (37.39)	40.55 (43.59)	
	28 ± 2	40.57 (46.61)	46.23 (63.24)	

*RMANCOVA analysis for the interaction of the variables treatment group and length of treatment.

^**1**^n = 21 for this treatment on day 28 ± 2.

A repeated measures model of covariance (RMANCOVA) analysis suggested a weak interaction between treatment and time, although this did not reach the significance level (p = 0.0750, Table 5). P-values for the effect of main factors (time, treatment) are not significant.

### Adverse events

A total of 56 AEs (cyclosporine A alone = 34; concurrent therapy = 22) were reported from 33 dogs. Approximately 76% of cases treated with cyclosporine A alone (n = 19/25) and 61% of cases treated with concurrent prednisolone (n = 14/23) showed at least one AE (difference not significant, p = 0.3532). No dogs died, were euthanized or stopped treatment due to AEs. None of the AEs reported was considered life-threatening, resulted in permanent disability or incapacity. All dogs made a complete recovery, except for five dogs (n = 3, cyclosporine A alone: conjunctivitis, luxating patella (both unlikely related to treatment) and otitis externa (unclassifiable); n = 2, concurrent therapy: abnormal laboratory value (unlikely related to treatment) and scaling, possibly related to treatment). Table 6 lists clinical signs reported by treatment and organized by a customized veterinary dictionary for drug related activities.

**Table 6:** Clinical signs reported and number of dogs affected per treatment group

Clinical sign*	**Cyclosporine A**^**1**^**(n = 25)**	**Cyclosporine A and prednisolone**^**1**^**(n = 23)**	**p-value**
Digestive tract disorders	20 (14)	12 (8)	0.1810
Abdominal pain	1	0	0.3589
Digestive tract hypermotility	0	1	0.3169
Flatulence	1	1	0.9762
Diarrhea	5 (5)	7 (6)	0.5815
Stomatitis	0	1	0.3169
Vomiting	13 (10)	2 (2)	**0.0116**
Renal and urinary disorders	4 (3)	7 (5)	0.3726
Cystitis	0	2 (2)	0.1441
Polyuria	1	2 (2)	0.5223
Uremia	0	1	0.3169
Urinary incontinence	1	0	0.3589
Urine abnormalities	2 (2)	2 (2)	0.9485
Skin and appendages disorders	10 (8)	3 (3)	0.1080
Bacterial skin infection	1	1	0.9762
Dermatitis	0	1	0.3169
Erosion	0	1	0.3169
Fungal skin infection	1	0	0.3589
Papilloma	1	0	0.3589
Pruritus	1	0	0.3589
Pyoderma	6	0	**0.0264**
Systemic disorders	2 (2)	13 (8)	**0.0190**
Abnormal test result	1	3 (3)	0.2717
Anorexia	0	1	0.3169
Lethargy	0	4 (4)	**0.0329**
Polydipsia	1	4 (4)	0.1384
Pyrexia	0	1	0.3169
Others			
Hyperactivity	1	0	0.3589
Tachypnea	1	1	0.9762
Musculoskeletal disorder	1	0	0.3589
Acid–base disorder	0	1	0.3169
Conjunctivitis	2 (2)	1	0.6226
Otitis externa	2 (2)	0	0.1798

*Several AEs had more than one clinical sign; total number of clinical signs reported: 81.

^1^The numbers in parentheses indicate the number of dogs showing the corresponding clinical sign. Dogs may have had more than one AE.

The most commonly reported clinical signs in the cyclosporine A alone group were associated with the digestive tract (47% of all reported clinical signs). A significantly lower incidence of vomiting was reported for the concurrent therapy group (2 reports in 2 dogs, vs. 13 reports in 10 dogs in the cyclosporine A alone group). Vomiting episodes were mild and typically only lasted for 1 to 2 days and withdrawal from the study was not required in any case. There was no significant differences between the groups in the incidence of skin and appendage disorders, with the exception of pyoderma, which was more frequently reported in the cyclosporine A alone group.

In contrast, systemic disorders were reported more frequently in the concurrent therapy group, in particular lethargy which was only observed in the concurrent therapy group (4 reports, p = 0.033). The reports of lethargy were of mild to moderate severity, started within the 20 days of the study (i.e. during the administration of prednisolone) and they resolved within 3 to 8 days following the end of prednisolone administration. Four reports of polydipsia in the combined treatment group were recorded, while only one polydipsia report occurred in the cyclosporine A group (p = 0.1384). Two dogs were reported with abnormal laboratory values in the cyclosporine A and prednisolone group consistent with the administration of prednisolone (elevated alkaline phosphatase; elevation of alkaline phosphatase, AST and ALT), while a third dog had elevated amylase, lipase, and cholesterol, in addition to pre-existing elevated creatinine and BUN values (indicating progression of a pre-existing condition). In the cyclosporine A group, one dog had a marginal BUN elevation, which recovered without intervention and was considered unlikely to be related to treatment.

### Hematology and clinical chemistry

No significant differences in mean baseline haematological or clinical chemistry values between the groups were observed at inclusion. Within each treatment group some differences were observed at the final assessment. A statistically significant increase in hematocrit and in the percentage of lymphocytes (in relation to total leukocytes) was observed in dogs treated with cyclosporine A alone Similarly, clinical chemistry values at study end revealed a statistically significant increase in cholesterol and a decrease in in ALT and AST in the cyclosporine A group, as well as an increase in albumin in the cyclosporine A and prednisolone group. An elevation in glucose was observed in both groups. However, all mean values for haematology and clinical chemistry were within reference range and the changes were considered clinically not significant. A summary of hematological and clinical chemistry changes is presented in Additional file 1: Table S2. There were no significant differences in any of the urine parameters measured.

## Discussion

Administration of cyclosporine has been shown to be effective and safe in the treatment of atopic dermatitis (recently reviewed by Palmeiro [15]). Here, we confirm that cyclosporine administered at 5 mg/kg once daily for 28 days administered concurrently with prednisolone administered for the first 20 days (1 mg/kg once daily for one week followed by 1 mg/kg once every other day for a further seven doses) was safe and resulted in a rapid reduction of pruritus in dogs treated for atopic dermatitis.

Multiple efficacy endpoints were evaluated in this study. Since owners consider pruritus the greatest burden to their dog’s quality of life [3], periodic evaluations of pruritus were primarily conducted by the owners, but also by the investigators. Owners reported a significant reduction of pruritus as early as 3–4 days after the initiation of treatment in dogs that received prednisolone concomitantly with cyclosporine A, and after 7–8 days in dogs treated with cyclosporine A alone. The sharper reduction in owner-reported pruritus in dogs in the concomitant treatment group (average reduction on day 7–8 of 60%) was in agreement with the overall assessment of clinical response. After 4 weeks of treatment, however, pruritus reduction was comparable between groups and similar to that reported elsewhere [6,16]. Furthermore, a similar proportion of dogs had either a ‘good’ or ‘excellent’ overall response to therapy on the final visit.

The CADESI-03 scoring system used here has been validated as a tool to assess change in skin lesions associated with atopic dermatitis [13]. In both treatment groups, CADESI-03 lesion scores were significantly lower both at day 14 ± 1 and 28 ± 2 when compared to pre-treatment scores, and there were no differences in lesion scores between groups at either visit. However, since the dispersion of the CADESI-03 scores for both treatments at each time point was considerable, it is possible that a larger data set would have allowed confirmation of a significant difference of CADESI-03 scores between groups at day 14 ± 1. Lesion scores after four weeks were remarkably similar, indicating that any potential difference between groups in CADESI-03 scores would be temporary (e.g. around day 14). Altogether this data shows that even though cyclosporine A administration leads to an improvement in atopic dermatitis signs 4 weeks after the initiation of therapy irrespective of the concurrent administration of steroids, dogs which had the benefit of an initial course of prednisolone had an accelerated reduction of pruritus and improvement of clinical score.

None of the AEs observed in this study were considered life-threatening, nor did they result in permanent disability or incapacity. The most commonly reported AEs following treatment with cyclosporine A were vomiting (25% overall prevalence) and diarrhea (15% overall prevalence) [6]. In this study, 10 out of 25 (40%) of the dogs included in the clinical safety analysis in the cyclosporine A group reported 13 vomiting events, in line with previous reports [5]. The reported vomiting was mild and improved spontaneously within a few days with continued use. In contrast, the prevalence of vomiting in the concurrent treatment group was significantly lower (9%). This observation was unexpected, and it is unclear why concurrent prednisolone would have reduced vomiting in dogs being treated with cyclosporine A. However, in view of the limited number of dogs included in this study, this observation may be incidental and should be interpreted with care. Additional studies may be warranted to investigate this potential effect.

The incidence of pyoderma in dogs treated with cyclosporine A alone was significantly higher than that in dogs treated with cyclosporine A and prednisolone. Pyoderma and otitis externa are often associated with atopic dermatitis and the observed incidence in this study may have been a result of the prohibition of the use of concurrent medications, notably antibiotics and otic preparations, usually used to manage these signs. Bacterial pyoderma is usually secondary to trauma. The dermal abrasion caused by scratching enables bacteria to colonize the skin and establish an infection. The data presented here suggests that the concomitant administration of prednisolone with cyclosporine A significantly reduces pruritus early in the course of treatment; therefore it is possible that dogs treated with cyclosporine A and prednisolone developed less secondary pyoderma due to the rapid reduction in pruritus and subsequent reduction of self-induced traumatic pyoderma.

Several studies have shown that treatment with elevated doses of glucocorticoids during prolonged periods of time can lead to immunosuppression [9]. For example, administration of 2.2-6.6 mg/kg of prednisolone twice daily for induction, followed by 1.1 to 2.2 mg/kg for maintenance to dogs can result in immunosuppressive effects, and increased risk of infection and poor wound healing are, in fact, among the most common side effects of glucocorticoid treatment in dogs [17]. In this study, a moderate dose of prednisolone was administered during a limited period and probably too short to result in a higher incidence of corticosteroid-related pyoderma.

The most frequently observed adverse events in the concurrent treatment group were polyuria, polydipsia and lethargy. These observations are consistent with reports of lethargy in dogs and cats being treated with glucocorticoids [17]. Polydipsia with secondary polyuria is a relatively common finding in dogs treated with glucocorticoids, and therefore this trend was expected [8]. All cases of polyuria and/or polydipsia and/or lethargy reported in dogs that had received cyclosporine A and prednisolone had resolved on, or before the final visit. Increases in liver enzymes, are also known side effects associated with the administration of corticosteroids [17]. In this study, three dogs were reported with enzyme elevations consistent with prednisolone therapy, two of which were transitory and clinically not significant, and a third case in which the abnormal values were likely to be related to a pre-existing condition. No further clinically significant changes in hematological, clinical chemistry or urinalysis parameters were observed.

## Conclusion

In conclusion, the data presented shows that the concurrent administration of a short course of prednisolone, at initiation of cyclosporine A therapy for the treatment of atopic dermatitis is well tolerated and efficacious in rapidly reducing pruritus. The combined treatment improves the dog’s overall clinical response assessed by the owner as soon as 3 to 4 days post treatment initiation. The adverse events observed were consistent with those expected for the individual veterinary medicinal products.

## Competing interests

RD, JC, IL, GS and ER are employees of Novartis Animal Health and declare no competing interests. RD is also affiliated to the University of Zurich, Switzerland. WR and EB were investigators in this trial and also received compensation as consultants to Novartis Animal Health. The authors declare that they have no competing interests.

## Authors’ contributions

RD was involved in the study design, in-life phase of the study and data analysis, and was responsible for interpretation of results and for writing the manuscript. JC and IL were involved in the study design, in-life phase of the study, interpretation of results, and were responsible for study management; GS was involved in the study design and was responsible for data analysis; ER was involved in the study design, in-life phase of the study and in the interpretation of the results. EB and WR equally contributed to the study design, in-life phase of the study and interpretation of results. All authors read and approved the final manuscript.

## Supplementary Material

Additional file 1 Overall clinical response assessed by the owner and summary of hematological and clinical chemistry changes per treatment group. Click here for file
