# Supplementary material for: Concurrent short-term use of prednisolone with cyclosporine A accelerates pruritus reduction and improvement in clinical scoring in dogs with atopic dermatitis
Source: BMC Vet Res. 2013 Sep 3;9:173. doi: 10.1186/1746-6148-9-173 (PMC3847128; doi:10.1186/1746-6148-9-173)
Supplement: Additional file 1 — Overall clinical response assessed by the owner and summary of hematological and clinical chemistry changes per treatment group. [file 1746-6148-9-173-S1.doc]

**Supporting data**

**Table S1. Overall clinical response (poor, moderate, good and excellent responders) assessed by the owner. Percentages are indicated between brackets.**

| **Days** | **Response** | **Number of responders (%)** | | **p-value*** |
| --- | --- | --- | --- | --- |
|  |  | **Cyclosporine A**  **(n=22)^1,2^** | **Cyclosporine A and prednisolone (n=22)^3^** |  |
| 3-4 | Poor | 12 (54.5) | 6 (27.3) | 0.0006 |
|  | Moderate | 10 (45.5) | 5 (22.7) |  |
|  | Good | 0 | 10 (45.5) |  |
|  | Excellent | 0 | 1 (4.5) |  |
| 7-8 | Poor | 6 (27.3) | 0 | 0.0001 |
|  | Moderate | 12 (54.5) | 4 (19) |  |
|  | Good | 4 (18.2) | 14 (66.7) |  |
|  | Excellent | 0 | 3 (14.3) |  |
| 10-11 | Poor | 4 (19) | 0 | 0.0007 |
|  | Moderate | 13 (61.9) | 5 (23.8) |  |
|  | Good | 4 (19) | 12 (57.1) |  |
|  | Excellent | 0 | 4 (19.1) |  |
| 14±1 | Poor | 2 (9.1) | 0 | 0.0024 |
|  | Moderate | 10 (45.5) | 3 (13.6) |  |
|  | Good | 10 (45.5) | 12 (54.5) |  |
|  | Excellent | 0 | 7 (31.8) |  |
| 17-18 | Poor | 3 (14.3) | 0 | 0.0010 |
|  | Moderate | 5 (23.8) | 1 (4.5) |  |
|  | Good | 13 (61.9) | 13 (59.1) |  |
|  | Excellent | 0 | 8 (36.4) |  |
| 21- 22 | Poor | 5 (25) | 1 (4.5) | 0.0151 |
|  | Moderate | 2 (10) | 5 (22.7) |  |
|  | Good | 12 (60) | 8 (36.4) |  |
|  | Excellent | 1 (5) | 8 (36.4) |  |
| 24-25 | Poor | 4 (20) | 1 (4.5) | 0.2202 |
|  | Moderate | 4 (20) | 3 (23.6) |  |
|  | Good | 10 (50) | 11 (50) |  |
|  | Excellent | 2 (10) | 7 (31.8) |  |
| 28±2 | Poor | 4 (19) | 2 (9.1) | 0.2178 |
|  | Moderate | 4 (19) | 1 (4.5) |  |
|  | Good | 11 (52.4) | 13 (59.1) |  |
|  | Excellent | 2 (9.5) | 6 (27.3) |  |

***** p-value: Fisher's Exact, two-tailed

**^1^** n=21 for this treatment on day 10-11, 17-18 and 28±2

**^2^** n=20 for this treatment group on day 21-22 and 24-35

**^3^** n=21 for this treatment on day 7-8, 10-11

**Table S2. Summary of hematological and clinical chemistry changes per treatment Group**

| **Parameter** | **Visit** | **Cyclosporine A** | | **Cyclosporine A and prednisolone** | | **Reference Range** |
| --- | --- | --- | --- | --- | --- | --- |
|  |  | **n** | **mean (s.d.)** | **n** | **mean (s.d.)** |  |
| Hematocrit  [%] | Baseline | 25 | 49.70 (5.63) ^1^ | 23 | 49.33 (5.74) | 36 - 60 |
|  | Day 28±2 | 24 | 51.91 (5.29) ^1^ | 22 | 48.98 (5.95) |  |
| Lymphocytes [%] | Baseline | 25 | 21.92 (6.79) ^1^ | 23 | 19.74 (7.53) | 12 - 30 |
|  | Day 28±2 | 24 | 26.83 (9.38) ^1,2^ | 22 | 20.32 (6.88) ^2^ |  |
| Cholesterol  [mg/dl] | Baseline | 25 | 202.03 (47.76) ^1^ | 23 | 237.94 (84.27) | 0 – 360 |
|  | Day 28±2 | 24 | 226.31 (69.24) ^1^ | 23 | 229.33 (75.61) |  |
| ALT [IU/L] | Baseline | 25 | 45.12 (32.94) ^1^ | 23 | 36.93 (14.07) | 0 - 122 |
|  | Day 28±2 | 24 | 43.42 (55.93) ^1^ | 23 | 58.26 (75.08) |  |
| AST [IU/L] | Baseline | 25 | 30.36 (7.39) ^1^ | 23 | 28.63 (13.10) | 0 - 66 |
|  | Day 28±2 | 24 | 25.71 (7.82) ^1^ | 23 | 32.04 (36.60) |  |
| Albumin  [g/L] | Baseline | 25 | 36.84 (3.29) | 23 | 34.96 (4.32) ^1^ | 27 - 44 |
|  | Day 28±2 | 24 | 36.58 (2.76) | 23 | 36.22 (3.40) ^1^ |  |
| CK [IU/L] | Baseline | 25 | 137.16 (69.22) ^1^ | 23 | 148.74 (104.92) ^1^ | 0 - 378 |
|  | Day 28±2 | 24 | 102.42 (63.86) ^1^ | 23 | 97.78 (47.15) ^1^ |  |
| Glucose [mg/dl] | Baseline | 25 | 96.04 (14.70) ^1^ | 23 | 92.74 (12.26) ^1^ | 70 - 138 |
|  | Day 28±2 | 24 | 102.96 (13.68) ^1^ | 23 | 100.96 (11.47) ^1^ |  |

**^1^** Significant differences within group (p ≤ 0.05)

**^2^** Significant differences between groups (p ≤ 0.05); n: number of samples analysed; s.d.: standard deviation
